# Association of Individual or Family History of Autoimmune Disease With Future Development of Type 1 Diabetes

**DOI:** 10.1002/dmrr.70110

**Published:** 2025-12-02

**Authors:** Nick Thomas, Bijay Vaidya, Richard David Leslie, Daniel Agardh, Richard Oram, Dana Dabelea, Arunjot Singh, Dimitrios Chantzichristos, Marian Rewers

**Affiliations:** ^1^ Institute of Biomedical and Clinical Science, University of Exeter Medical School Devon UK; ^2^ Department of Endocrinology Royal Devon and Exeter Hospital, University of Exeter Medical School Exeter UK; ^3^ Centre for Immunobiology Blizard Institute, Queen Mary University of London London UK; ^4^ Unit of Celiac Disease and Diabetes, Department of Clinical Sciences Lund University Lund Sweden; ^5^ Lifecourse Epidemiology of Adiposity & Diabetes Center University of Colorado Anschutz Medical Campus Aurora Colorado USA; ^6^ Division of Gastroenterology, Hepatology and Nutrition Children's Hospital of Philadelphia Philadelphia Pennsylvania USA; ^7^ Department of Pediatrics Perelman School of Medicine at the University of Pennsylvania Philadelphia Pennsylvania USA; ^8^ Department of Internal Medicine and Clinical Nutrition Institute of Medicine, Sahlgrenska Academy, University of Gothenburg Gothenburg Sweden; ^9^ Department of Endocrinology‐Diabetes‐Metabolism Sahlgrenska University Hospital Gothenburg Sweden; ^10^ Barbara Davis Center for Diabetes University of Colorado Anschutz Medical Campus Aurora Colorado USA

**Keywords:** adrenal insufficiency, autoimmune disease, coeliac disease, risk factors, thyroid disease, type 1 diabetes

## Abstract

Timely diagnosis of type 1 diabetes (T1D), especially in high‐risk populations, is crucial for preventing serious health complications. T1D is a chronic progressive autoimmune disease that has presymptomatic stages that can be identified through the detection of islet autoantibodies. Given that T1D is associated with other autoimmune diseases, having either those diseases or a family history of them will represent a risk of T1D. From a search of the literature conducted in August 2024, we review here the evidence for the risk of either T1D or the development of T1D in association with other autoimmune diseases or a family history of those diseases. Increased risk of subsequent T1D development was identified for individuals with autoimmune diseases, including coeliac disease, autoimmune thyroid disease, autoimmune Addison's disease, juvenile idiopathic arthritis, primary biliary cholangitis, ulcerative colitis, vitiligo, and myasthenia gravis. Increased prevalence of diabetes‐associated autoantibody positivity was found among non‐diabetic individuals with coeliac and autoimmune thyroid diseases compared with individuals without these autoimmune diseases. Increased risk of T1D was also found for individuals with a family history of autoimmune diseases, including coeliac disease, thyroid disease, Addison's disease, rheumatoid arthritis, systemic lupus erythematosus, Sjögren's syndrome, autoimmune liver disease, pernicious anaemia, inflammatory bowel disease, multiple sclerosis, and granulomatosis with polyangiitis. This review highlights how certain individuals at risk of T1D can be identified to offer them islet autoantibody screening and, thereby, early detection of T1D.

**Abbreviations**
APS‐1: autoimmune polyendocrine syndrome type 1
APS‐2: autoimmune polyendocrine syndrome type 2
CI: confidence interval
DIPP: Diabetes Prediction and Prevention
GADA: glutamic acid decarboxylase autoantibodies
HLA: human leucocyte antigen
HR: hazard ratio
IA‐2A: insulinoma‐associated antigen 2 autoantibodies
IAA: insulin autoantibodies
ICA: islet cell autoantibodies
IPEX: X‐linked immunodysregulation, polyendocrinopathy, and enteropathy
IQR: interquartile range
IRR: incidence rate ratio
JIA: juvenile idiopathic arthritis
MMN: multifocal motor neuropathy
MS: multiple sclerosis
NR: not reported
OR: odds ratio
RA: rheumatoid arthritis
RR: relative risk
SLE: systemic lupus erythematosus
T1D: type 1 diabetes
TEDDY: The Environmental Determinants of Diabetes in the Young
ZnT8A: zinc transporter 8 autoantibodies

## 1 Introduction

Type 1 diabetes (T1D) is a chronic, progressive autoimmune disease characterised by irreversible destruction of insulin‐producing beta cells in the pancreas, which typically leads to reliance on exogenous insulin to regulate blood glucose [1, 2]. Clinical disease (stage 3 T1D) onset may occur at any age [3, 4, 5, 6] and is preceded by early presymptomatic disease stages (stages 1 and 2 T1D), which can be identified through the detection of ≥ 2 islet autoantibodies [7, 8]. For children who develop ≥ 2 islet autoantibodies, the lifetime risk of progressing to stage 3 T1D has been found to approach 100%, with the 5‐, 10‐, and 15‐year risk being 43.5%, 69.7%, and 84.2%, respectively [7, 9]. The 10‐year risk of progressing to stage 3 T1D for children with single islet autoantibody positivity has been found to be lower (14.5%), but for those with insulinoma‐associated antigen 2 autoantibody (IA‐2A) positivity, the risk is significantly higher (40.5%) than for those with glutamic acid decarboxylase autoantibody (GADA) positivity (12.9%) or insulin autoantibody (IAA) positivity (13.1%) [9]. For adults with ≥ 2 islet autoantibodies, the 5‐year risk of progressing to stage 3 has been found to be 15% [10].

Identification of risk factors for T1D is important for understanding who to prioritise for islet autoantibody screening. Individuals with a family history of T1D or with certain genetic risk factors are at increased risk of T1D compared with the general population [11, 12]. Individuals with other autoimmune diseases or with a family history of them may also have increased T1D genetic susceptibility [13, 14, 15]. In general, autoimmune diseases tend to cluster in individuals and in families [15, 16, 17]. Across studies worldwide, T1D has been associated (clustered) with various other (concomitant) autoimmune diseases [18, 19, 20]. Diseases clustering with T1D include, but are not limited to, autoimmune thyroid disease, coeliac disease, autoimmune Addison's disease, autoimmune gastritis, juvenile idiopathic arthritis (JIA), and rheumatoid arthritis (RA) [13, 21, 22, 23, 24, 25, 26, 27, 28]. Such associations may be due in part to shared genetic risk factors [29, 30, 31, 32]. For example, specific HLA haplotypes (DR3‐DQ2 and DR4‐DQ8) have been found to confer increased risk for T1D and coeliac disease [33]. T1D that occurs along with autoimmune thyroid disease or autoimmune Addison's disease may be a part of autoimmune polyendocrine syndrome type 2 (APS‐2) [34, 35]. T1D may also occur as a part of autoimmune polyendocrine syndrome type 1 (APS‐1) and is a main manifestation of X‐linked immunodysregulation, polyendocrinopathy, and enteropathy (IPEX), which are both monogenic disorders [34, 35]. Although several studies have described an association between T1D and other autoimmune diseases, only some of these studies provide insight into the sequence of the development of the autoimmune diseases or the risk of subsequent development of T1D in individuals with other autoimmune diseases.

Understanding which individuals with other autoimmune diseases are at increased risk of T1D is important for developing screening programs for early presymptomatic T1D. Benefits of such early detection of T1D include preventing life‐threatening acute disease presentation (i.e., diabetic ketoacidosis), reducing the need for hospitalisation at clinical onset, and improving glycaemic control subsequently [36, 37, 38]. Avoiding diabetic ketoacidosis is important not only because it is associated with substantial morbidity and mortality in the short‐term but also because it is associated with poor long‐term glycaemic control [39, 40]. Early detection also allows for the potential use of disease‐modifying therapy to delay the onset of clinical T1D [41]. With an early diagnosis, clinicians can offer better education and monitoring, which may improve outcomes and quality of life [36, 37, 38]. The objective of this review is to describe the available evidence on the development of T1D following other autoimmune diseases and the risk of T1D in individuals with a family history of other autoimmune diseases. Previous reviews have described the association between T1D and other autoimmune diseases [42, 43, 44]; the present review adds to the literature by focussing on evidence regarding whether the presence of other autoimmune diseases signifies a risk of *subsequent* development of T1D. This information is important for informing strategies (clinical guidance) of identifying individuals at risk and of screening for early presymptomatic T1D and for understanding the areas where more research is needed.

## 2 Methods

A search of PubMed for articles, conducted on August 16, 2024, included terms related to various autoimmune diseases (coeliac, thyroid, Hashimoto's, hyperthyroidism, hypothyroidism, thyroiditis, Graves', Addison's, primary adrenal insufficiency, myasthenia gravis, rheumatoid arthritis, juvenile idiopathic arthritis, juvenile rheumatoid arthritis, rheumatic joint, autoimmune hepatitis, primary biliary, autoimmune liver, inflammatory bowel, Crohn's, ulcerative colitis, psoriasis, vitiligo, pernicious anaemia, multiple sclerosis, and systemic lupus erythematosus) combined with the term *type 1 diabetes* and phrases that limited the search to articles on the sequence of development of T1D and other autoimmune diseases. Titles and abstracts were first reviewed to determine articles for potential inclusion in the literature review. A total of 548 articles were identified from the PubMed search, and of these, 36 articles were included based on eligibility criteria. The details of the literature search and eligibility criteria are in the Supporting Information S1: Appendix. An additional 15 articles that met eligibility criteria were also included; these were identified through citations in eligible articles or were previously known to the authors. Findings on islet autoantibody positivity reflect different assays and thresholds for positivity; therefore, caution should be exercised in comparisons between studies.

## 3 Findings of the Literature Search

### 3.1 Studies Investigating Multiple Autoimmune Diseases

#### 3.1.1 T1D Development Following Other Autoimmune Diseases

Findings by Conrad et al. suggest that individuals with several other autoimmune diseases are at increased risk of developing subsequent T1D before age 20 [13]. This study used a longitudinal database of electronic health records of individuals in the UK (*N* = 22,009,375) to examine the incidence rate of T1D diagnosed at ≤ 19 years of age in individuals with other autoimmune diseases [13]. Compared with the general population, a higher incidence of subsequent T1D diagnosis was found for individuals with Addison's disease (incidence rate ratio [IRR] = 11.7; 95% CI: 3.8–36.2), Graves' disease (IRR = 9.9; 95% CI: 5.6–17.5), myasthenia gravis (IRR = 7.6; 95% CI: 1.1–53.6), primary biliary cholangitis (IRR = 7.5; 95% CI: 1.1–53.4), Hashimoto's thyroiditis (IRR = 6.1; 95% CI: 4.6–8.1), coeliac disease (IRR = 4.1; 95% CI: 2.8–6.2), and vitiligo (IRR = 2.0; 95% CI: 1.3–3.0) (Table 1).

**TABLE 1:** Risk of subsequent T1D development in individuals with another autoimmune disease.

Article	Autoimmune disease	Study design	Findings
Conrad et al. 2023 [13]	Multiple diseases were investigated	Electronic health records of individuals in the UK	A higher incidence rate of T1D (diagnosed at ≤ 19 years) was found among individuals with the following autoimmune diseases compared with the general population: Addison's disease (adjusted IRR = 11.7; 95% CI: 3.8–36.2) Graves' disease (adjusted IRR = 9.9; 95% CI: 5.6–17.5) Myasthenia gravis (adjusted IRR = 7.6; 95% CI: 1.1–53.6), Primary biliary cholangitis (adjusted IRR = 7.5; 95% CI: 1.1–53.4), Hashimoto's thyroiditis (adjusted IRR = 6.1; 95% CI: 4.6–8.1) Coeliac disease (adjusted IRR = 4.1; 95% CI: 2.8–6.2) Vitiligo (adjusted IRR = 2.0; 95% CI: 1.3–3.0)
Ludvigsson et al. 2006 [45]	Coeliac disease	Study of Swedish registers	Children and adolescents with coeliac disease diagnosis before 20 years (*N* = 9243) had an increased risk of subsequent T1D before 20 years compared with controls (*N* = 45,680) (HR = 2.4; 95% CI: 1.9–3.0)
Canova et al. 2016 [46]	Coeliac disease	Retrospective cohort study	Children and young adults with coeliac disease did *not* show a significantly increased risk of subsequent T1D compared with matched controls (HR: 2.50; 95% CI: 0.94–6.66)
Sun et al. 2024 [47]	Inflammatory bowel disease	Swedish cohort study	Individuals ≤ 28 years of age with inflammatory bowel disease had a higher risk of developing subsequent T1D than controls (adjusted HR: 1.58; 95% CI: 1.27–1.95). The findings were driven by individuals with ulcerative colitis (adjusted HR = 2.02; 95% CI: 1.51–2.70)
Lee et al. 2020 [28]	JIA	Cohort study using claims database	Children and adolescents with JIA (*N* = 15,210) showed an increased risk of subsequent T1D compared with matched controls (*N* = 76,050) (adjusted HR = 1.81; 95% CI: 1.03–3.17)
Jacobsen et al. 2024 [48]	MS	Study of Danish registers	Individuals with MS did *not* show a significantly increased risk of subsequent T1D compared with controls (adjusted HR: 1.60; 95% CI: 0.98–1.40 [sic]; *p* = 0.07)

Abbreviations: HR, hazard ratio; IRR, incidence rate ratio; JIA, juvenile idiopathic arthritis; MS, multiple sclerosis; RR, relative risk; T1D, type 1 diabetes.

Additional studies that reported the prevalence of other autoimmune diseases in individuals at the time of T1D diagnosis provide evidence that T1D may follow the clinical onset of other autoimmune diseases (Table 2A) [22, 49]. For example, in a Swedish register study of children with T1D (*N* = 15,188) and matched controls (*N* = 74,210), children with T1D had higher rates of coeliac disease (1.8% vs. 0.5%), thyroid disease (0.6% vs. 0.2%), and vitiligo (0.2% vs. 0.1%) at T1D onset compared with controls at study entry [49].

**TABLE 2:** (A) Prevalence of other autoimmune diseases at T1D diagnosis and (B) percentage of individuals diagnosed with T1D before, at the same time as, and after another autoimmune condition.

A. Prevalence of other autoimmune diseases at T1D diagnosis			
Article	Autoimmune disease	Study design	Findings
Samuelsson et al. 2024 [49]	Multiple diseases were investigated	Study of Swedish registers	Children with T1D had higher rates of coeliac disease (1.8% vs. 0.5%), autoimmune thyroid disease (0.6% vs. 0.2%), and vitiligo (0.2% vs. 0.1%) at T1D onset compared with controls at study entry
Triolo et al. 2011 [22]	Multiple diseases were investigated	Single‐centre study in the US	3.1% (15/491) of children with T1D had autoimmune thyroid disease at T1D diagnosis, 2.9% (14/491) had coeliac disease, and 0.2% (1/491) had autoimmune Addison's disease
Lindgren et al. 2024 [50]	Coeliac disease	Cohort study in Sweden	1.9% (99/5295) of children and adolescents with T1D had coeliac disease before their T1D diagnosis, and 3.9% (204/5295) were diagnosed with coeliac disease within 11.9 months after their T1D diagnosis
Bianchi et al. 2016 [51]	Coeliac disease	Retrospective database study in Italy	0.5% (8/1563) of children and adolescents with T1D were diagnosed with coeliac disease before T1D
Cerutti et al. 2004 [52]	Coeliac disease	Cohort study in Italy	0.8% (34/4322) of children and adolescents with T1D received a diagnosis of coeliac disease before that of T1D, and 0.7% (31/4322) received a diagnosis of coeliac disease at the same time as T1D
Valerio et al. 2002 [53]	Coeliac disease	Single‐centre study in Italy	2.1% (8/383) of children and adolescents with T1D were diagnosed with T1D after coeliac disease
Pocecco et al. 1995 [54]	Coeliac disease	Survey study of 19 Italian paediatric diabetes centres	0.3% (14/4514) of individuals with T1D were diagnosed with coeliac disease before T1D
Kaspers et al. 2004 [55]	Coeliac disease	Multicentre retrospective cohort study in Germany and Austria	In children and adolescents with T1D, coeliac disease was diagnosed before T1D in 0.07% (13/19,796)
Greco et al. 2011 [56]	Thyroid disease	Single‐centre retrospective study in Italy	1.9% (9/470) of children and adults with T1D had an onset of Graves' disease before T1D onset, and 0.9% (4/470) had an onset at the same age as T1D

B. Percentage of individuals with another autoimmune disease diagnosed with T1D before, at the same time as, or after the other autoimmune disease					
Article	Autoimmune disease	Study design	T1D before the other autoimmune disease	T1D at the same time as the other autoimmune disease	T1D after the other autoimmune disease
Schirru et al. 2024 [31]	Coeliac disease	Sardinian cohort study	6.6% (54/822)		2.7% (22/822)
Pallotta et al. 2024 [80]	Coeliac disease	Prospective study of Italian adults	0.9% (8/920)		2.3% (21/920)
Cruz et al. 2007 [83]	Thyroid disease	Retrospective medical record review in Brazil	0.8% (2/254)	NR	2.4% (6/254)
Fichna et al. 2016 [84]	Addison's disease	Cross‐sectional single‐centre study in Poland	3.6% (5/140)	0.7% (1/140)	5.7% (8/140)
Fichna et al. 2010 [85]	Addison's disease	Cross‐sectional study in Poland	3.5% (3/85)	1.2% (1/85)	2.4% (2/85)
Betterle et al. 2013 [86]	Addison's disease	Single‐centre retrospective study in Italy	8.1% (40/492)	1.4% (7/492)	1.6% (8/492)
Schenck et al. 2018 [88]	JIA	German database study	0.3% (34/12,269)	NR unknown timing: 0.03% (4/12,269)	0.2% (20/12,269)

Abbreviations: JIA, juvenile idiopathic arthritis; NR, not reported; T1D, type 1 diabetes.

#### 3.1.2 Presence of Islet Autoantibodies in Individuals With Other Autoimmune Diseases

A study by Pietropaolo et al. examined the prevalence of positivity for islet autoantibodies (GADA and IA‐2A) in children and adults with autoimmune thyroid disease, systemic lupus erythematosus (SLE), RA, and primary biliary cirrhosis (without clinical T1D) [57]. The study found positivity for at least one islet autoantibody in 1.8% (1/53) of individuals with autoimmune thyroid disease, 3.7% (1/27) with SLE, 3.1% (1/32) with RA, and 5.5% (3/55) with primary biliary cirrhosis compared with 1.1% (3/280) of controls. None, however, were positive for both GADA and IA‐2A (Table 3).

**TABLE 3:** Prevalence of islet autoantibody positivity in individuals with another autoimmune disease (without clinical T1D^a^).

Article	Autoimmune disease	Study design; autoantibodies examined	Findings
Pietropaolo et al. 1998 [57]	Multiple diseases were investigated	Study of individuals at outpatient clinics; GADA and IA‐2A	Positivity for at least 1 islet autoantibody was found in 1.8% (1/53) of children and adults with autoimmune thyroid disease, 3.7% (1/27) with SLE, 3.1% (1/32) with RA, and 5.5% (3/55) with primary biliary cirrhosis compared with 1.1% (3/280) of controls. None of these individuals was positive for both GADA and IA‐2A
Tiberti et al. 2017 [58]	Coeliac disease	IAA, GADA, IA‐2A, ZnT8A	A higher prevalence of positivity for islet autoantibodies was found in children and adolescents with coeliac disease (5.5% [29/529]) compared with healthy controls (1.1% [3/264]; *p* = 0.002)
Légeret et al. 2023 [59]	Coeliac disease	Retrospective study in Switzerland; GADA, IA‐2A, IAA, ZnT8A	*No* difference found in the prevalence of islet autoantibody positivity in serum samples of 95 children with coeliac disease and 199 matched controls with functional abdominal disease (1.9% vs. 2.3%, respectively)
Ghozzi et al. 2021 [60]	Coeliac disease	Retrospective study in Tunisia; GADA, IA‐2A, ZnT8A	A higher prevalence of islet autoantibody positivity was found in adults with coeliac disease (12.5% [10/80]) compared with controls (1.1% [1/90]; *p* = 0.003)
Putarek et al. 2023 [61]	Thyroid disease	Single‐centre study in Croatia; GADA, IA‐2A, ICA	A higher prevalence of islet autoantibody positivity was found in children and adolescents with an autoimmune thyroid disease (10.6%; 17/161) than in controls (1.9%; 3/155; *p* = 0.002)
Pilia et al. 2011 [62]	Thyroid disease	Single‐centre study of Sardinian children; GADA, IA‐2A	Of the children and adolescents with autoimmune thyroiditis, 8.1% (19/236) had islet autoantibody positivity at the time of diagnosis compared with 4.1% (39/949) of controls (*p* = 0.017)
Hawa et al. 2006 [20]	Thyroid disease	Single‐centre study in Cameroon; GADA, IA‐2A	Of 18 adults with thyrotoxicosis, 22.2% (4/18) were positive for GADA compared with 5.6% (2/36) of controls, 27.8% (5/18) were positive for IA‐2A compared with 2.8% (1/36) of controls, and 16.6% (3/18) were positive for both
Bilbao et al. 2004 [63]	MS	Study in southern Europe; GADA, IA‐2A, and autoantibodies against carboxypeptidase H	*No* evidence was found of increased prevalence of islet autoantibody positivity in serum samples of 49 individuals with MS compared with known general population levels

^a^ ‘Clinical T1D’ refers to a diagnosis of stage 3 T1D.

Abbreviations: GADA, glutamic acid decarboxylase autoantibodies; IA‐2A, insulinoma‐associated antigen 2 autoantibodies; IAA, insulin autoantibodies; ICA, islet cell autoantibodies; JIA, juvenile idiopathic arthritis; MS, multiple sclerosis; RA, rheumatoid arthritis; SLE, systemic lupus erythematosus; T1D, type 1 diabetes; ZnT8A, zinc transporter 8 autoantibodies.

#### 3.1.3 T1D in Individuals With a Family History of Other Autoimmune Diseases

Several studies have found evidence of an increased risk of T1D in individuals with a family history of autoimmune diseases (Table 4) [14, 64, 65, 66]. A retrospective mother‐child cohort study (*N* = 1,288,347) in Taiwan found a higher risk of childhood‐onset T1D in children whose mothers had Hashimoto's thyroiditis (adjusted hazard ratio [HR] = 3.73; 95% CI: 1.70–8.15), inflammatory bowel disease (adjusted HR = 2.00; 95% CI: 1.07–3.76), or any autoimmune disease (adjusted HR = 1.55; 95% CI: 1.16–2.08) [64]. A database study in Sweden found an increased standardised incidence ratio for T1D for individuals with a parent with Addison's disease (2.40; 95% CI: 1.40–3.86), coeliac disease (2.73; 95% CI: 1.98–3.67), hyperthyroidism (1.86; 95% CI: 1.65–2.08), hypothyroidism (2.35; 95% CI: 1.78–3.06), pernicious anaemia (3.09; 95% CI: 2.00–4.57), primary biliary cirrhosis (3.63; 95% CI: 2.02–6.00), RA (2.12; 95% CI: 1.90–2.36), SLE (2.04; 95% CI: 1.48–2.75), ulcerative colitis (1.23; 95% CI: 1.06–1.42), and Wegener's granulomatosis (now known as granulomatosis with polyangiitis) (2.12; 95% CI: 1.39, 3.08) compared with individuals whose parents did not have autoimmune diseases [14]. The standardised incidence ratio was also increased for individuals with a sibling with autoimmune Addison's disease (3.91; 95% CI: 1.42–9.70), coeliac disease (1.92; 95% CI: 1.13–3.25), or hyperthyroidism (1.83; 95% CI: 1.02–3.24) [14].

**TABLE 4:** Risk of T1D in individuals with a family history of another autoimmune disease.

Article	Autoimmune disease	Study design	Findings
Yen et al. 2023 [64]	Multiple diseases were investigated	Retrospective mother‐child cohort study in Taiwan	A higher risk of childhood‐onset T1D was found for children whose mothers had Hashimoto's thyroiditis (adjusted HR = 3.73; 95% CI: 1.70–8.15), inflammatory bowel diseases (adjusted HR = 2.00; 95% CI: 1.07–3.76), or any autoimmune disease (adjusted HR = 1.55; 95% CI: 1.16–2.08)
Hemminki et al. 2009 [14]	Multiple diseases were investigated	Database study in Sweden	An increased SIR^a^ for T1D was found for individuals with a parent with the following autoimmune diseases compared with individuals without relatives with the autoimmune diseases: Addison's disease (2.40; 95% CI: 1.40–3.86) Coeliac disease (2.73; 95% CI: 1.98–3.67) Hyperthyroidism (1.86; 95% CI: 1.65–2.08) Hypothyroidism (2.35; 95% CI: 1.78–3.06) Pernicious anaemia (3.09; 95% CI: 2.00–4.57) Primary biliary cirrhosis (3.63; 95% CI: 2.02–6.00) RA (2.12; 95% CI: 1.90–2.36) SLE (2.04; 95% CI: 1.48–2.75) Ulcerative colitis (1.23; 95% CI: 1.06–1.42) Wegener's granulomatosis (granulomatosis with polyangiitis) (2.12; 95% CI: 1.39–3.08) The SIR^a^ was also increased for individuals with a sibling with: Addison's disease (3.91; 95% CI: 1.42–9.70) Coeliac disease (1.92; 95% CI: 1.13–3.25) Hyperthyroidism (1.83; 95% CI: 1.02–3.24)
Tait et al. 2004 [65]	Multiple diseases were investigated	Study of individuals in the UK and Ireland in a DNA repository	Individuals with T1D had a significantly higher prevalence of mothers and fathers with the following autoimmune diseases compared with the general population: RA Vitiligo Pernicious anaemia
Sipetić et al. 2002 [66]	Multiple diseases were investigated	Case‐control study in Belgrade	Individuals with T1D had a higher prevalence of a positive family history of the following autoimmune diseases compared with controls: Thyroid diseases (26.3% vs. 11.4%, *p* < 0.001) Rheumatic diseases (21.9% vs. 12.4%, *p* < 0.05) Coeliac disease and Crohn's disease (5.7% vs. 0.5%, *p* < 0.05)
Emilsson et al. 2015 [67]	Coeliac disease	Study of a Swedish cohort and Swedish registers	First‐degree relatives (*N* = 84,648) of individuals with coeliac disease had an increased risk of developing T1D compared with matched controls (adjusted HR = 1.65; 95% CI: 1.41–1.93)
Neuhausen et al. 2008 [68]	Coeliac disease	Questionnaire study of individuals from US and Canada	First‐degree relatives (*N* = 1272) of individuals with coeliac disease were found to have an increased risk of T1D compared with the general population (standardised ratio = 3.2; 95% CI: 1.7–5.3)
Skov et al. 2022 [69]	Thyroid disease	Study of Swedish registers	Individuals with an identical twin with Hashimoto's thyroiditis had an increased risk of T1D compared with individuals with a twin without Hashimoto's (adjusted risk ratio: 8.0; 95% CI: 3.4–18.9). Risk of T1D was *not* increased in individuals with a fraternal twin with Hashimoto's (adjusted risk ratio: 1.8; 95% CI: 0.7–4.3)
Hemminki et al. 2010 [70]	Thyroid disease	Swedish database study	Offspring of parents with Graves' disease had an increased risk of T1D compared with individuals without relatives with Graves' disease (SIR^a^ = 2.08, 95% CI: 1.66–2.56 in women; SIR^a^ = 2.17, 95% CI: 1.78–2.63 in men)
Thomsen et al. 2020 [71]	Addison's disease	Study of Swedish registers	Individuals with a first‐degree relative with autoimmune Addison's disease had a higher risk of T1D compared with individuals without a first‐degree relative with Addison's disease (SIR^a^: 2.38, 95% CI: 1.72–3.16)
Kuo et al. 2017 [72]	RA	Taiwanese national database study	Risk of T1D was higher in individuals with a first‐degree relative with RA compared with the general population (adjusted RR = 1.96; 95% CI: 1.54–2.48)
Kuo et al. 2015 [73]	Sjögren's disease	Taiwanese national database study	Risk of T1D was higher in individuals with a first‐degree relative with Sjögren's disease compared with the general population (adjusted RR = 1.97; 95% CI: 1.29–3.02)
Kuo et al. 2015 [74]	SLE	Taiwanese national database study	Risk of T1D was higher in individuals with a first‐degree relative with SLE compared with the general population (adjusted RR = 1.68; 95% CI: 1.22–2.32)
Ulff‐Møller et al. 2017 [75]	SLE	Danish national database study	Risk of T1D in first‐degree relatives of individuals with SLE was slightly increased compared with individuals without relatives with SLE (HR = 1.23; 95% CI: 1.01–1.48)
Nielsen et al. 2006 [76]	MS	Study of Danish registers	Risk of T1D was increased (adjusted RR = 1.51; 95% CI: 1.14–2.00) for offspring of individuals with MS but *not* for siblings of individuals with MS (adjusted RR = 1.15; 95% CI: 0.58–2.30)
Marrosu et al. 2002 [77]	MS	Single‐centre study in Italy	Siblings of individuals with MS who had another first‐ or second‐degree relative with MS (*N* = 450) were found to be at increased risk of T1D compared with the general population (*p* = 0.02) and compared with siblings without other relatives with MS (*p* = 0.03)
Cats et al. 2012 [78]	MMN	Case‐control study	T1D was significantly more prevalent in first‐degree relatives of individuals with MMN (1.2%) compared with first‐degree relatives of controls (0.2%, *p* = 0.002)

^a^ SIR is the ratio of the number of observed cases to the number of expected cases.

Abbreviations: HR, hazard ratio; MMN, multifocal motor neuropathy; MS, multiple sclerosis; RA, rheumatoid arthritis; RR, relative risk; SIR, standardised incidence ratio; SLE, systemic lupus erythematosus; T1D, type 1 diabetes.

An additional study examined the prevalence of autoantibody positivity for T1D, coeliac disease, and autoimmune thyroid disease in children and adolescents (who were not known to have these diseases) with a first‐degree relative with these diseases [15]. A higher prevalence of autoantibody positivity for any of these diseases was found for children with a first‐degree relative with T1D, coeliac disease, or autoimmune thyroid disease than for children without a first‐degree relative with these diseases (odds ratio [OR] = 2.52; 95% CI: 1.83–3.47). Furthermore, children with a first‐degree relative with T1D, coeliac disease, or autoimmune thyroid disease had a higher frequency of diagnosis for these diseases following screening than children without a first‐degree relative with these diseases (OR = 3.61; 95% CI: 1.82–7.18). Approximately half (52.4%; 33/63) of children who tested positive for autoantibodies were positive for autoantibodies of a disease different from their first‐degree relative. Although this study did not specifically report on T1D, it suggests that family history of other autoimmune diseases may be important in considering the risk for T1D, coeliac disease, and autoimmune thyroid disease. Consistent with this, a large UK and USA T1D twin study found that the best‐fitting model of the combined cohorts included additive genetic and unique environmental factors with a heritability estimate for thyroid autoimmunity of 69% [79].

**FIGURE 1:**
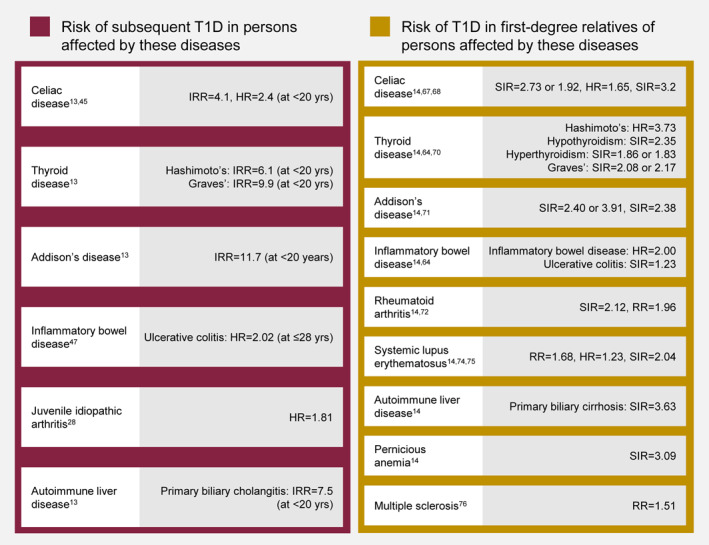
Risk of subsequent T1D development in individuals with other autoimmune diseases (left) and risk of T1D in first‐degree relatives of individuals with other autoimmune diseases (right). Risk is relative to the general population or a control group. HR, hazard ratio; IRR, incidence rate ratio; RR, relative risk; SIR, standardised incidence ratio; T1D, type 1 diabetes; yrs, years.

Further evidence regarding the development of T1D following another autoimmune disease or with a family history of another autoimmune disease is described below for specific autoimmune diseases. Summaries of the findings of this review are presented in Tables 1, 2, 3, 4 and in Figure 1.

### 3.2 Coeliac Disease

#### 3.2.1 T1D Development Following Coeliac Disease

At least one study in coeliac disease has found an increased risk of T1D development following diagnosis of coeliac disease (Table 1) [45]. In a study of Swedish registers by Ludvigsson et al., children and adolescents (*N* = 9243) with a hospital inpatient diagnosis of coeliac disease were found to have an increased risk of subsequent T1D diagnosis before 20 years of age compared with matched controls (*N* = 45,680) (HR = 2.4; 95% CI: 1.9–3.0). Coeliac disease was also associated with an increased risk of subsequent diabetic ketoacidosis (HR = 2.3; 95% CI: 1.4–3.9) [45]. This study demonstrated an increased risk of T1D for those with coeliac disease. However, cases of coeliac disease may have been missed due to the lack of regular screening for relevant autoantibodies. In a retrospective cohort study of children and young adults with coeliac disease and matched controls, 0.5% (6/1215) of those with coeliac disease and 0.2% (12/6048) of controls developed subsequent T1D. The HR for subsequent T1D development was 2.50 (95% CI: 0.94–6.66), which was similar in magnitude to the HR reported by Ludvigsson et al. but was not statistically significant [46].

Other studies have reported the proportion of individuals with coeliac disease who were diagnosed with T1D after (and before) coeliac disease (Table 2B) [31, 80]. For example, in a cohort study including 822 individuals with coeliac disease, 2.7% (22/822) received a T1D diagnosis after their coeliac disease diagnosis, and 6.6% (54/822) received a T1D diagnosis before or at the same time as their coeliac disease diagnosis [31]. In addition, several studies have documented the prevalence of coeliac disease before a T1D diagnosis among individuals with T1D (Table 2A) [50, 51, 52, 53, 54, 55].

#### 3.2.2 Presence of Islet Autoantibodies in Individuals With Coeliac Disease

A couple of studies have found a higher prevalence of positivity for islet autoantibodies in individuals with coeliac disease compared with controls (Table 3) [58, 60]. Tiberti et al. found a higher prevalence of islet autoantibody positivity (IAA, GADA, IA‐2A, or zinc transporter 8 autoantibodies [ZnT8A]) in children and adolescents with coeliac disease (5.5% [29/529]) compared with healthy controls (1.1% [3/264]; *p* = 0.002). [58] In contrast, a retrospective study by Légeret et al. found no difference in the prevalence of islet autoantibody positivity (GADA, IA‐2A, IAA, or ZnT8A) in serum samples of 95 children with coeliac disease and 199 matched controls with functional abdominal disease (without a diagnosis of coeliac disease) and negative for coeliac disease–associated autoantibodies (1.9% vs. 2.3%, respectively) [59]. In a retrospective study, Ghozzi et al. examined serum samples of 80 adults with coeliac disease (60 with newly diagnosed coeliac disease) without known T1D and 90 healthy controls for GADA, IA‐2A, and ZnT8A. Of the 80 patients with coeliac disease, 12.5% (10/80) showed positivity for GADA compared with 1.1% (1/90) of controls (*p* = 0.003) [60]. Only 1 adult with coeliac disease showed positivity for the other islet autoantibodies; this individual had simultaneous positivity for all three islet autoantibodies. None of the controls showed positivity for IA‐2A or ZnT8A [60].

#### 3.2.3 Islet and Coeliac Disease–Associated Autoantibodies in Individuals at Risk for Both T1D and Coeliac Disease

Studies have also examined the sequence of autoantibody positivity for coeliac disease and T1D in those at risk of both diseases. The Environmental Determinants of Diabetes in the Young (TEDDY) study is a prospective, multinational (US, Finland, Germany, Sweden) birth cohort study that included children at genetic risk for both T1D and coeliac disease [81]. Serum was collected every 3 months from 3 to 48 months of age and at least every 6 months thereafter and analysed for islet autoantibodies (GADA, IA‐2A, and IAA). Testing for coeliac disease–associated autoantibodies was conducted annually beginning at 24 months of age; if a sample was positive, then previously collected samples from that child were tested to find the first negative sample in time closest to the time‐point of seroconversion [33]. 90 children developed both islet autoantibodies and coeliac disease–associated autoantibodies. Of these, 27% (24/90) developed coeliac disease–associated autoantibodies before islet autoantibodies, 6% (5/90) developed islet autoantibodies and coeliac disease autoantibodies simultaneously, and 68% (61/90) developed islet autoantibodies before coeliac disease autoantibodies [33]. In a cohort of the prospective Diabetes Prediction and Prevention (DIPP) study, children with genetic risk for both T1D and coeliac disease (*N* = 2052) were followed to determine the order in which autoantibodies for T1D and coeliac disease developed. Children were observed every 3 months until 2 years of age and every 6 months thereafter or at 3, 6, 12, 18, and 24 months of age and every 12 months thereafter. If a child developed positivity for islet cell autoantibodies (ICA), positivity for IAA, GADA, and IA‐2A were assessed in all previous and future samples [82]. Of the 19 children who developed both islet and coeliac disease–associated autoantibodies, 42.1% (8/19) developed coeliac disease–associated autoantibodies first, 15.8% (3/19) developed the autoantibodies at the same time, and 42.1% (8/19) developed islet autoantibodies first [82]. In a Chinese nationally representative sample of 4671 adults with diabetes aged ≥ 20 years, IA‐2A were associated with coeliac disease–associated autoantibodies (OR: 19.05; 95% CI: 3.14, 115.6) [18].

#### 3.2.4 T1D in Individuals With a Family History of Coeliac Disease

Data suggest that a family history of coeliac disease is a risk factor for T1D (Table 4) [67, 68]. Based on data from Swedish health care registries, first‐degree relatives (*N* = 84,648) of individuals with coeliac disease had an increased risk of developing T1D compared with matched controls (adjusted HR = 1.65; 95% CI: 1.41–1.93). In contrast, spouses of individuals with coeliac disease did not show an increased risk of T1D [67]. In a study based on questionnaire data, first‐degree relatives (*N* = 1272) of individuals with coeliac disease were found to have an increased risk of T1D compared with the general population (standardised ratio = 3.2; 95% CI: 1.7–5.3) [68].

### 3.3 Autoimmune Thyroid Disease

#### 3.3.1 T1D Development Following Autoimmune Thyroid Disease

At least one study reported the proportion of individuals with autoimmune thyroid disease who were diagnosed with T1D after (and before) thyroid disease (Table 2B) [83]. Cruz et al. conducted a retrospective medical records review of 254 individuals with autoimmune thyroid disease (*n* = 150 with Graves' orbitopathy, mean age: 41.8 ± 13.0 years; *n* = 104 without Graves' orbitopathy, mean age: 36.9 ± 14.0 years), most of whom had hyperthyroidism. T1D was diagnosed following thyroid disease in 2.4% (6/254) and was diagnosed before thyroid disease in 0.8% (2/254). Only 1 patient with orbitopathy developed T1D following thyroid disease [83]. At least one study has reported the prevalence of autoimmune thyroid disease (Graves' disease) before T1D diagnosis in individuals with T1D (Table 2A) [56].

#### 3.3.2 Presence of Islet Autoantibodies in Individuals With Autoimmune Thyroid Disease

Studies have reported a higher prevalence of positivity for islet autoantibodies in individuals with autoimmune thyroid disease (Table 3) [20, 61, 62]. Putarek et al. examined islet autoantibodies (GADA, IA‐2A, and ICA) in a study of 161 children and adolescents with autoimmune thyroid disease (autoimmune thyroiditis: *n* = 127; Graves' disease: *n* = 34) and 155 controls without autoimmune conditions in Croatia [61]. Individuals with autoimmune thyroid disease were found to have a higher frequency of islet autoantibody positivity (GADA or IA‐2A) (10.6%; 17/161) compared with the control group (1.9%; 3/155; *p* = 0.002). (Notably, one adolescent with islet autoimmunity had clinical T1D at the time of evaluation and another child had impaired glucose tolerance.) The frequency was found to be higher in the individuals with autoimmune thyroiditis (11.8%; *p* = 0.001) but not Graves' disease (5.9%; *p* = 0.19). Five individuals with autoimmune thyroid disease and islet autoantibodies developed T1D during the 6‐year follow‐up. In another study, Pilia et al. examined the presence of islet autoantibodies (GADA and IA‐2A) in 236 children and adolescents with autoimmune thyroiditis without diabetes and 949 healthy controls. Of the children and adolescents with autoimmune thyroiditis, 8.1% (19/236) had islet autoantibody positivity at the time of diagnosis of autoimmune thyroiditis compared with 4.1% (39/949) of controls (*p* = 0.017). After 1 year, 10 children were reevaluated for autoantibody positivity; seven of 10 children remained positive. Over 2 years of follow‐up, two children who were positive for islet autoantibodies developed T1D [62].

#### 3.3.3 T1D in Individuals With a Family History of Autoimmune Thyroid Disease

Risk of T1D has been examined for individuals with a family history of Hashimoto's thyroiditis and Graves' disease (Table 4) [69, 70]. In a Swedish study, individuals with an identical twin with Hashimoto's had an increased risk of T1D compared with individuals with a twin without Hashimoto's (adjusted risk ratio: 8.0; 95% CI: 3.4–18.9); however, risk of T1D was not increased in individuals with a fraternal twin with Hashimoto's (adjusted risk ratio: 1.8; 95% CI: 0.7–4.3) [69]. A separate Swedish database study found an increased risk of T1D in offspring of parents with Graves' disease compared with individuals without relatives with Graves' disease. The standardised incidence ratio for T1D among offspring of parents with Graves' disease was 2.08 (95% CI: 1.66–2.56) in women and 2.17 (95% CI: 1.78–2.63) in men [70].

### 3.4 Autoimmune Addison's Disease

#### 3.4.1 T1D Development Following Autoimmune Addison's Disease

A few studies of autoimmune Addison's disease reported the proportion of individuals with autoimmune Addison's disease who were diagnosed with T1D after (and before) autoimmune Addison's disease (Table 2B) [84, 85, 86, 87]. A cross‐sectional study in Poland of individuals with autoimmune Addison's disease admitted to a single centre found that T1D was diagnosed after autoimmune Addison's disease in 5.7% (8/140), at the same time as autoimmune Addison's disease in 0.7% (1/140), and before autoimmune Addison's disease in 3.6% (5/140) [84]. A single‐centre retrospective study of individuals with Addison's disease in Italy found that T1D was diagnosed after autoimmune Addison's disease in 1.6% (8/492), at the same time as autoimmune Addison's disease in 1.4% (7/492), and before autoimmune Addison's disease in 8.1% (40/492) [86]. For comparison, a nationwide Swedish register study found that 0.34% (105/30,790) of adults with T1D were later diagnosed with autoimmune Addison's disease [87].

#### 3.4.2 T1D in Individuals With a Family History of Autoimmune Addison's Disease

A study of Swedish registers found an increased risk of T1D in individuals with a first‐degree relative with autoimmune Addison's disease compared with individuals without a first‐degree relative with Addison's disease (Table 4). The standardised incidence ratio of T1D among individuals with a first‐degree relative with Addison's disease was 2.38 (95% CI: 1.72–3.16) [71].

### 3.5 Inflammatory Bowel Disease

#### 3.5.1 T1D Development Following Inflammatory Bowel Disease

A cohort study in Sweden including 20,314 individuals with inflammatory bowel disease ≤ 28 years of age (Crohn's disease: *n* = 7277; ulcerative colitis: *n* = 10,112; unclassified inflammatory bowel disease: *n* = 2925) and 99,200 matched controls found that individuals with inflammatory bowel disease had a higher risk of developing subsequent T1D than controls (adjusted HR = 1.58; 95% CI: 1.27–1.95; Table 1) [47]. A higher risk of subsequent T1D was found when specifically examining individuals with ulcerative colitis (adjusted HR = 2.02; 95% CI: 1.51–2.70) but not individuals with Crohn's disease or unclassified inflammatory bowel disease. When compared to siblings without inflammatory bowel disease, individuals with inflammatory bowel disease still showed an increased risk of subsequent T1D (adjusted HR = 1.44; 95% CI: 0.97–2.15).

### 3.6 Juvenile Idiopathic Arthritis, Rheumatoid Arthritis, Sjögren's Syndrome, or Systemic Lupus Erythematosus

#### 3.6.1 T1D Development Following JIA

A claims‐based study found increased risk of subsequent T1D diagnosis in children and adolescents with JIA (*N* = 15,210) compared with matched healthy controls (*N* = 76,050) (adjusted HR = 1.81; 95% CI: 1.03–3.17; Table 1) but not compared with children with asthma (adjusted HR = 1.48; 95% CI: 0.86–2.56) [28]. The children with asthma were chosen as a comparison group because they were likely to regularly visit healthcare providers and be similarly exposed to steroids. The incidence rate of T1D per 100,000 person‐years was 44 (95% CI: 28–70) for children with JIA, 22 (95% CI: 17–28) for healthy children, and 29 (95% CI: 23–36) for children with asthma [28].

Another study reported the proportion of individuals with JIA who developed T1D after (and before) JIA (Table 2B) [88]. In a study of 12,269 individuals ≤ 20 years of age with JIA in a German national rheumatologic database, 58 also had T1D, and the timing of the onset of both diseases was available for 54. Of those with JIA and T1D, 37% developed T1D after JIA symptoms and 63% developed T1D before JIA. Of those who developed T1D after JIA, JIA symptoms preceded the onset of T1D by a mean of 40 months [88].

#### 3.6.2 T1D in Individuals With a Family History of RA, SLE, or Sjögren's Syndrome

Studies of individuals enrolled in the Taiwan National Healthcare Insurance system in 2010 (*N* = 23,658,577) found that the risk of T1D was higher in individuals with a first‐degree relative with RA, SLE, or Sjögren's compared with the general population (Table 4) [72, 73, 74]. Specifically, the adjusted relative risk of T1D was 1.96 (95% CI: 1.54–2.48) in individuals with a first‐degree relative with RA, 1.68 (95% CI: 1.22–2.32) in individuals with a first‐degree relative with SLE, and 1.97 (95% CI: 1.29–3.02) in individuals with a first‐degree relative with Sjögren's [72, 73, 74]. A separate nationwide cohort study of individuals (*N* = 5,237,319) in the Danish Civil Registration System found that the risk of T1D in first‐degree relatives of individuals with SLE was slightly increased compared with individuals without relatives with SLE (HR = 1.23; 95% CI: 1.01–1.48) [75].

### 3.7 Multiple Sclerosis

#### 3.7.1 T1D Development Following MS

A study of Danish registers examined individuals with a multiple sclerosis (MS) diagnosis (*N* = 13,376) and matched controls (*N* = 66,880), followed for a median of 8.3 years. The risk of subsequent T1D in individuals with MS was not significantly different from controls (adjusted HR: 1.60; 95% CI: 0.98–1.40 [sic]; *p* = 0.07; Table 1) [48].

#### 3.7.2 Presence of Islet Autoantibodies in Individuals With MS

A small study in southern Europe found no evidence of increased prevalence of islet autoantibody positivity (GADA, IA‐2A, or carboxypeptidase H autoantibodies) in serum samples of 49 individuals with MS compared with known general population levels (Table 3) [63].

#### 3.7.3 T1D in Individuals With a Family History of MS

Despite the lack of an association between MS and T1D in individuals, a couple of studies have found that there may be a slightly increased risk of T1D for individuals with a family history of MS (Table 4). A study of Danish registers examined the risk of T1D in offspring and in siblings of individuals with MS (*N* = 14,771 offspring and siblings). The adjusted relative risk of T1D was increased for offspring (1.51 [95% CI: 1.14–2.00]) but not for siblings (1.15 [95% CI: 0.58–2.30]) of individuals with MS (adjusted for first‐degree relatives with T1D) [76]. In a single‐centre interview study in Italy, siblings of individuals with MS who had another first‐ or second‐degree relative with MS (*N* = 450) were found to be at increased risk of T1D compared with the general population (2.2% vs. 0.54%; *p* = 0.02) and compared with siblings without other relatives with MS (2.2% vs. 0.67%; *p* = 0.03) [77].

## 4 Age of Onset of Type 1 Diabetes and Associated Autoimmune Diseases

The age of onset of autoimmune diseases may be associated with their sequence of development within an individual. T1D may occur at any age throughout the lifespan [3, 4, 5, 6]. In a study that investigated T1D onset in the US in children and young adults (aged 0–19), the peak incidence of T1D diagnosis occurred at age 10 [89]. Other studies that have investigated the incidence of T1D in children and adults have found that over half of new cases of T1D occur in adults [90, 91]. One modelling study estimated that in 2021, 62% of new cases of T1D were in individuals ≥ 20 years [91] Given that T1D may occur at any age, it could be postulated that T1D could be preceded by any other associated autoimmune disease. However, it may be more likely for T1D to develop after other autoimmune diseases that typically occur early in life.

The incidence of various autoimmune diseases across the human lifespan was reported by Conrad et al. based on electronic health record data in the UK [13]. For coeliac disease, new diagnoses were found to occur throughout most of the lifespan, beginning at an early age, and with a median age at diagnosis of 45 years (interquartile range [IQR]: 26, 62). Where T1D or coeliac disease developed in childhood, peak onset age appeared earlier in coeliac disease than in T1D [13]. This is consistent with the finding that there is an increased risk of subsequent T1D development among those with coeliac disease [13, 45]. Other studies in children and adolescents with T1D found that among those who develop both T1D and coeliac disease, the T1D diagnosis preceded the coeliac disease diagnosis more often than it followed the coeliac disease diagnosis [50, 51, 52, 53, 54, 55]. However, the practice of beginning regular screening for coeliac disease once a child is diagnosed with T1D may have influenced these findings.

In contrast with coeliac disease, incidence was found to increase across the lifespan for Hashimoto's thyroiditis, Graves' disease, Addison's disease, and RA [13]. The median (IQR) age at diagnosis was numerically higher for RA (65 years [IQR: 53, 77]) than for Hashimoto's thyroiditis (58 years [IQR: 45, 72]), Graves' disease (59 years [IQR: 43, 75]), and Addison's disease (58 years [IQR: 41, 73]) [13]. The high age of onset for RA may explain in part why T1D has been found to be a risk factor for subsequent development of RA, but RA was not found to be a risk factor for subsequent development of T1D [13]. Notably, factors other than the typical age of onset of each autoimmune disease may also play a role in the sequence of autoimmune disease development within individuals.

## 5 Expert Opinion: Screening for Early Presymptomatic Type 1 Diabetes in Individuals With Autoimmune Disease or a Family History of Other Autoimmune Diseases

Based on the evidence, individuals with certain autoimmune diseases or a family history of them may have an increased risk of T1D and may benefit from screening for islet autoantibodies to identify early presymptomatic T1D, as described below. Recommendations and guidance on screening for early presymptomatic T1D and subsequent metabolic monitoring have been previously published [92, 93, 94, 95]. Clinicians should screen for early presymptomatic T1D at the onset of a personal or family history of an autoimmune disease that increases risk for T1D and at < 3 years of age if family history precedes birth. Further research is needed to determine the optimal frequency of screening and when screening can be discontinued in individuals who have negative screening results.

### 5.1 Coeliac Disease

Individuals with coeliac disease have been found to have an increased risk of subsequent T1D development (at < 20 years of age) compared with the general population or with controls [13, 45]. Consistent with this, a higher prevalence of islet autoantibody positivity has been found in children and adolescents with coeliac disease and in adults with coeliac disease compared with controls [58, 60]. An increased risk of T1D has also been found in individuals with a first‐degree relative with coeliac disease [14, 67, 68]. These findings suggest that individuals with coeliac disease or with a family history of coeliac disease are at increased risk of developing T1D and should be considered for early presymptomatic T1D screening.

### 5.2 Autoimmune Thyroid Disease

At least one study has found an increased risk of T1D development (at < 20 years of age) compared with the general population in individuals with Hashimoto's thyroiditis or Graves' disease [13]. Studies have also found a higher prevalence of islet autoantibody positivity in children and adolescents with autoimmune thyroiditis and in adults with thyrotoxicosis (notably, the aetiology of thyrotoxicosis was unclear from the study) [20, 61, 62]. Other studies have reported an increased risk of T1D in individuals with a first‐degree relative with autoimmune thyroid disease (for both Hashimoto's thyroiditis and Graves' disease) [14, 64, 70]. These findings suggest that children and young adults with Hashimoto's thyroiditis or Graves' disease are at increased risk of T1D and should be considered for screening for early presymptomatic T1D. Further studies are needed to assess the risk of T1D in individuals with a family history of these diseases.

### 5.3 Autoimmune Addison's Disease

One study showed an increased risk of T1D development (at < 20 years) in individuals with Addison's disease compared with the general population [13]. Studies have also shown an increased risk of T1D in individuals with a first‐degree relative with autoimmune Addison's disease [14, 71]. The most recent guidelines for primary adrenal insufficiency suggest annual screening for T1D [96]. Those recommendations are based on a low level of evidence. Further studies are needed to better understand the risk of T1D in individuals with autoimmune Addison's disease and when to screen for both diseases.

### 5.4 Inflammatory Bowel Disease

A large study in individuals ≤ 28 years of age found an increased risk of subsequent T1D in those with inflammatory bowel disease, specifically ulcerative colitis. Risk was not found to be increased for those with Crohn's disease or unclassified inflammatory bowel disease [47]. In addition, children of parents with inflammatory bowel disease and ulcerative colitis specifically have been found to have an increased risk of T1D [14, 64]. Further studies are needed to better understand the risk of T1D in individuals with inflammatory bowel disease.

### 5.5 Juvenile Idiopathic Arthritis, Rheumatoid Arthritis, or Sjögren's Syndrome

One large study found an increased risk of T1D compared with controls in children and adolescents with JIA [28]. Other studies have shown an increased risk of T1D in individuals with a family history of RA or with first‐degree relatives with Sjögren's [14, 65, 72, 73]. Further studies are needed to better understand the risk of T1D in individuals with JIA and RA. Individuals with a family history of RA should be considered for screening for early presymptomatic T1D.

### 5.6 Systemic Lupus Erythematosus

Studies have found that individuals with first‐degree relatives with SLE are at an increased risk of T1D [14, 73, 75]. Furthermore, a Mendelian randomisation analysis—an analysis technique that uses genetic variants associated with potential risk factors to overcome confounding and reverse causation in determining causal relationships [97]—found a bidirectional causal association between SLE and T1D. Individuals with a family history of SLE should be considered for screening for early presymptomatic T1D. Further studies are needed to understand the risk of T1D in individuals with SLE.

### 5.7 Multiple Sclerosis

Certain HLA‐DR/DQ genotypes that confer a predisposition to MS have been found to be protective against the development of T1D [77]. Nevertheless, there are studies showing an association between the two diseases. One study found a slightly increased risk of T1D in individuals with a parent with MS [76]. Another study found an increased risk of T1D in individuals with two relatives with MS [77]. Further studies are needed to better understand the risk of T1D in individuals with a family history of MS.

### 5.8 Autoimmune Liver Disease

Conrad et al. found an increased risk of subsequent T1D development in individuals with primary biliary cholangitis [13]. Furthermore, Hemminki et al. found an increased risk of T1D in individuals with a parent with primary biliary cirrhosis [14]. Further research is needed to better understand the risk of T1D associated with autoimmune liver disease.

### 5.9 Pernicious Anaemia

Hemminki et al. found an increased risk of T1D in individuals with a parent with pernicious anaemia [14]. Further research is needed to better understand the risk of T1D associated with this disorder.

## 6 Conclusions

Evidence suggests that individuals with specific autoimmune diseases or with a family history of certain autoimmune diseases are at increased risk of future development of T1D. A significant increased risk of subsequent T1D development has been found in individuals with coeliac disease, autoimmune thyroid disease, Addison's disease, ulcerative colitis, juvenile idiopathic arthritis, myasthenia gravis, primary biliary cholangitis, and vitiligo. Studies of family history have shown a significant increased risk of T1D in individuals with a first‐degree relative with coeliac disease, autoimmune thyroid disease, Addison's disease, RA, Sjögren's, SLE, MS, pernicious anaemia, inflammatory bowel disease, granulomatosis with polyangiitis, autoimmune liver disease, and vitiligo. Individuals at increased risk of T1D could benefit from screening for early presymptomatic T1D to prevent severe disease morbidity at clinical onset of T1D and improve long‐term glycaemic control. Early detection of T1D allows for prompt initiation of therapy and follow‐up. By diagnosing early, clinicians can offer better education and monitoring, improving outcomes and quality of life. Additionally, early intervention can minimise hospitalisations and may reduce the burden on health care systems. This is notable for individuals with autoimmune Addison's disease and T1D as they face an increased risk of hypo‐ or hyperglycemia (with or without diabetic ketoacidosis) as well as adrenal crisis compared to those with either condition alone [98]. Further research is needed to better understand the frequency and impact of screening for early presymptomatic T1D in individuals with other autoimmune diseases or with a family history of other autoimmune diseases.

## Author Contributions

N.T., B.V., R.D.L., D.A., R.O., D.D., A.S., D.C., and M.R. conceived and critically reviewed and edited the manuscript. All authors have read and approved the final manuscript.

## Funding

Medical writing was funded by Sanofi.

## Conflicts of Interest

NT: Support for attending meetings and/or travel from Young Diabetologists and Endocrinologists Forum and European Association for the Study of Diabetes (EASD) travel award.

BV: Leadership or fiduciary role for British Thyroid Association (secretary; 2021–2024), Programme Committee of the UK Society for Endocrinology (member; 2019–2023), Executive Committee of the European Thyroid Association (member; 2018–2022), *Thyroid Research* journal (Editor, 2015‐)

RDL: Participation on a data safety monitoring board and advisory board for Diamyd.

DA: Consulting fees from Sanofi; payment or honoraria from Sanofi; support for attending meetings from Sanofi; participation on data safety monitoring board or advisory board for Sanofi.

RO: Research grant or contracts from Randox and Sanofi; royalties and licence from Randox; consulting fees from Sanofi, Provention Bio, and Janssen; payment or honoraria from Sanofi and Novo Nordisk; participation on data safety monitoring board or advisory board for Sanofi.

DD: The author has nothing to report.

AS: The author has nothing to report.

DC: The author has nothing to report.

MR: Grants or contracts from Sanofi US; consulting fees from Sanofi US and Janssen R&D; payment or honoraria from Provention Bio.

For all authors, medical writing support for this manuscript was funded by Sanofi.

## Supporting information


Supporting Information S1

## Data Availability

Data presented in this article can be found in the references cited.
