# Supplementary material for: Association of Individual or Family History of Autoimmune Disease With Future Development of Type 1 Diabetes
Source: Diabetes Metab Res Rev. 2025 Dec 2;42(1):e70110. doi: 10.1002/dmrr.70110 (PMC12670487; doi:10.1002/dmrr.70110)
Supplement: Supplementary file 1 — Supporting Information S1 [file DMRR-42-e70110-s001.docx]

**Supplementary Appendix**

**Association of Individual or Family History of Autoimmune Disease With Future Development of Type 1 Diabetes**

Nick Thomas, Bijay Vaidya, Richard David Leslie, Daniel Agardh, Richard Oram, Dana Dabelea, Arunjot Singh, Dimitrios Chantzichristos, Marian Rewers

**LITERATURE SEARCH**

**Search Date:** August 16, 2024

**No date limit**

**PubMed Search Terms:**

((celiac) OR (thyroid) OR (Hashimoto's) OR (hyperthyroidism) OR (hypothyroidism) OR (thyroiditis) OR (Graves') OR (Addison's) OR (“primary adrenal failure”) OR (“primary adrenal insufficiency”) OR (“myasthenia gravis”) OR (“rheumatoid arthritis”) OR (“juvenile idiopathic arthritis”) OR (“juvenile rheumatoid arthritis”) OR ("rheumatic joint") OR (“autoimmune hepatitis”) OR (“primary biliary”) OR (“autoimmune liver”) OR (“inflammatory bowel”) OR (Crohn’s) OR (“ulcerative colitis”) OR (psoriasis) OR (vitiligo) OR (“pernicious anemia”) OR (“multiple sclerosis”) OR (“systemic lupus erythematosus”))

AND (“type 1 diabetes”)

AND ((“risk of type 1 diabetes”) OR (“risk of subsequent type 1 diabetes”[tiab:~0]) OR (“risk of developing type 1 diabetes”[tiab:~0]) OR (“incidence of type 1 diabetes”) OR (“incident type 1 diabetes”) OR (“screening for type 1 diabetes”[tiab:~0]) OR (“comorbid autoimmune”) OR (“comorbid type 1 diabetes”) OR (“susceptibility to type 1 diabetes”[tiab:~0]) OR ("other autoimmune") OR ("additional autoimmune") OR ("other AID") OR ("additional AID") OR (“likelihood of type 1 diabetes”[tiab:~0]))

**Number of articles identified from the PubMed search:** 548 articles

**Note:** [tiab:~0] was used for terms not appearing in the PubMed phrase index to search for the items in quotes next to each other in any order.

**ARTICLE ELIGIBILITY CRITERIA**

To be included in the review, articles had to be research studies on humans and be written in English. Articles also had to describe at least 1 of the following:

- Risk of type 1 diabetes (T1D) development following the development of another autoimmune disease
- Prevalence of another autoimmune disease before or at the diagnosis of T1D
- Proportion of individuals with another autoimmune disease (with or without a diagnosis of T1D) who had a diagnosis of T1D after the other autoimmune disease
- Prevalence of islet autoantibody positivity (eg, insulin autoantibodies [IAA], glutamic acid decarboxylase autoantibodies [GADA], insulinoma-associated antigen 2 autoantibodies [IA-2A], zinc transporter 8 autoantibodies [ZnT8A], and islet cell autoantibodies [ICA]) in individuals with other autoimmune diseases but without a diagnosis of clinical T1D compared with controls
- Among individuals who developed both islet autoantibodies and other autoimmune-specific autoantibodies, the proportion of individuals who developed islet autoantibodies second
- Risk of T1D in individuals with a family history of another autoimmune disease compared with controls

Articles that were case reports or review articles were excluded. Other articles were excluded for any of the following reasons:

- The sequence of T1D diagnosis and diagnosis of the other autoimmune disease was not reported
- The prevalence of positivity for islet autoantibodies was reported in individuals with another autoimmune disease, but at least some of the individuals also had a diagnosis of clinical T1D. (For these articles, the sequence of autoimmune disease development was unclear)
- T1D and type 2 diabetes were grouped together
